# Lifestyle Behaviors, Depression, and Anxiety Among Individuals Living in Canada During the COVID-19 Pandemic

**DOI:** 10.1177/15598276221102097

**Published:** 2022-05-30

**Authors:** Mario Simjanoski, Taiane de Azevedo Cardoso, Bianca Wollenhaupt-Aguiar, Bianca Pfaffenseller, Raquel B. De Boni, Vicent Balanzá-Martínez, Benicio N. Frey, Luciano Minuzzi, Flavio Kapczinski

**Affiliations:** Neuroscience Graduate Program, McMaster University, Hamilton, Canada; Department of Psychiatry and Behavioral Neurosciences, 62703 McMaster University, Hamilton, Canada; Department of Psychiatry and Behavioral Neurosciences, 62703 McMaster University, Hamilton, Canada; Institute of Scientific and Technological Communication and Information in Health (ICICT), 37903 Oswaldo Cruz Foundation (FIOCRUZ), Rio de Janeiro, Brazil; Teaching Unit of Psychiatry and Psychological Medicine, Department of Medicine, 16781 University of Valencia, CIBERSAM, Valencia, Spain; Department of Psychiatry and Behavioral Neurosciences, 62703 McMaster University, Hamilton, Canada; Women’s Health Concerns Clinic and Mood Disorders Program, St. Joseph’s Healthcare, Hamilton, ON, Canada; Department of Psychiatry and Behavioral Neurosciences, 62703 McMaster University, Hamilton, Canada; Neuroscience Graduate Program, McMaster University, Hamilton, Canada; Department of Psychiatry and Behavioral Neurosciences, McMaster University, Hamilton, Canada; Instituto Nacional de Ciência e Tecnologia Translacional em Medicina (INCT-TM), Porto Alegre, Brazil

**Keywords:** mental health, short multidimensional lifestyle inventory evaluation—confinement, lockdown, pandemic, North America, lifestyle medicine

## Abstract

The aim of our study was to investigate the association between lifestyle behaviors and symptoms of depression and anxiety during the COVID-19 pandemic in Canada. A web survey was conducted between July 3–August 3, 2020, across Canada. The main outcomes considered were a positive screening for depression, as evaluated by the PHQ-2 and positive screening for anxiety, as evaluated by the GAD-7. Lifestyle behaviors were assessed using the Short Multidimensional Lifestyle Inventory Evaluation—Confinement (SMILE-C), an instrument adapted for lifestyle behaviors during the COVID-19 pandemic. The total sample size included 404 participants, of which 24.3% had a positive screen for depression, 20.5% for anxiety, and 15.5% for both. We found significant differences in SMILE-C scores between individuals with a positive and individuals with a negative screen for depression (*P* < .001). Likewise, there were significant differences in SMILE-C scores between individuals with a positive and individuals with a negative screen for anxiety (*P* < .001). We found an association between unhealthy lifestyle behaviors and symptoms of depression and anxiety during the COVID-19 lockdown in Canada. The findings highlight the importance of lifestyle medicine (LM) education and targeted lifestyle interventions to promote healthy behaviors and help reduce the burden of mental disorders.

“This study revealed an association between self-reported unhealthy lifestyle behaviors and positive screenings for depression and anxiety symptoms during the COVID-19 lockdown in Canada.”

## Introduction

The global coronavirus disease 2019 (COVID-19) pandemic has taken a toll on the mental well-being of individuals from various regions in the world. The unpredictable longevity of the pandemic has forced many countries to apply various restrictions on everyday activities for their citizens, including Canada.^1^ Since the onset of the COVID-19 pandemic, people have been advised to limit in-person social interactions and adjust their day-to-day routines in order to contain the spread of the novel severe acute respiratory syndrome coronavirus 2 (SARS-CoV-2) virus. In addition to the documented physical health consequences of the virus, the sudden lifestyle changes have resulted in rising uncertainty, fear, stress, and higher levels of mental health challenges among the general population.^2^ During this time, online tools and web surveys have been widely used to collect data and investigate the psychological well-being of the general population.^3,4^ Among the most pronounced mental health challenges are the increased prevalence of depression and anxiety, with recent reports combining data from multiple countries indicating symptoms of anxiety in 31.9% and symptoms of depression in 33.7% of the general population.^5,6^

As in other countries, people in Canada have also been affected by the COVID-19 pandemic in this sense; the percentage of high anxiety among Canadians has quadrupled (5% to 20%), while the percentage of severe symptoms of depression has more than doubled during this period (4% to 10%).^7^ Another study in Canada reported very high prevalence of symptoms of anxiety (47.2%) and depression (44.1%),^8^ demonstrating the serious level of concern in Canada and beyond. In addition, the high mental health burden is significantly present among some of the most overwhelmed essential workers in Canada, critical care nurses, with 57% of them reporting mild to severe symptoms of depression and 67% reporting mild to severe symptoms of anxiety.^9^

Seeking to contain the transmission of the SARS-CoV-2 virus, many people had to change their everyday routines and lifestyles relative to before the onset of the pandemic. Diet, physical activity, sleep, substance use, and many other essential lifestyle behaviors are strongly linked to symptoms of common mental disorders, such as depression and anxiety.^10-12^ In accordance with surges of COVID-19, the provincial governments of Canada have continuously adjusted the lockdown measures required to contain the spread of the virus since March of 2020. The restrictive measures put in place have led to Canadians becoming less physically active, having more sleep difficulties, fewer social interactions, and other negative lifestyle changes detrimental to physical and psychological well-being.^7,13,14^ The relationship between these factors with mental health symptoms and disorders has been further emphasized during the COVID-19 pandemic, with the home-confinement measures introduced around the world leading to lifestyle changes in North Africa, Western Asia, and Europe.^15,16^ The urgency to suddenly change many aspects of these lifestyle behaviors has led to serious negative impacts on people’s overall mental well-being. In Brazil, a study found a positive association between the presence of psychological symptoms (e.g., depression, anxiety, and stress) and social isolation variables (e.g., days of isolation, loneliness, and social distancing) adopted for containment of the wide-spreading virus.^17^ Furthermore, unhealthy lifestyle patterns during COVID-19 were found to increase the likelihood of depression and anxiety among essential workers in Brazil and Spain.^18^

The significant focus on different lifestyle behaviors during this period stems from the scope of lifestyle medicine (LM), an area of medicine that studies how daily habits and behaviors impact short- and long-term health and quality of life, and uses evidence-based approaches to prevent and treat the progression of chronic diseases.^19,20^ According to the American College of LM, LM is particularly focused on 6 lifestyle domains: diet, physical activity, avoiding substance use, sleep, social relationships, and stress management.^21^ These domains are deemed to be “modifiable factors,” meaning, targeting prevention or improvement of unhealthy daily lifestyle behaviors could help in reducing the risk for development of poor mental health and other chronic diseases.^19,22^ Maintaining a healthy lifestyle can be a protective factor for many physical and mental health challenges, especially during times of adversity, such as the COVID-19 pandemic.

Although recent studies have reported increased prevalence of depression and anxiety in Canada during the pandemic, the role of unhealthy lifestyle habits on people’s mental well-being, associated with the changes in daily routines during this period, has not been investigated. In this sense, the aim of this study was to examine the association between several lifestyle behaviors and the presence of symptoms of depression and anxiety during the COVID-19 pandemic in a Canadian sample.

## Methods

### Study Design

A cross-sectional web survey was conducted from July 3 to August 3, 2020, across all regions in Canada. The web survey was created using SurveyGizmo®,^23^ and included questions regarding demographics, COVID-19 experience, lifestyle behaviors, substance use, self-rated health (SRH) status and previously diagnosed conditions, as well as current symptoms of depression and anxiety. The usability and technical functionality of the survey were tested prior to the start of recruitment.

### Ethical Aspects

The study was approved by the Hamilton Integrated Research Ethics Board (HiREB) under protocol #10870.

### Study Population and Recruitment

A convenience sample of individuals living in Canada that were 18 and older, had access to the internet, and agreed to participate in the study after reviewing the informed consent form was recruited to complete the web survey. The weblink to the online survey was available on a Facebook page (fb.me/lifestyleandcovid19) created for this project. Advertising boost to promote the questionnaire was prepared using multiple key words (i.e., healthy lifestyles, sad, happiness, fear, physical exercise, stress, and well-being). In addition, the study was shared through other social media outlets, such as Instagram (instagram.com/lifestyleandcovid19), Twitter (twitter.com/LifestyleCovid), WhatsApp, and email. Individuals who reported they had already previously completed the online questionnaire were excluded.

### Independent Variables

Demographic information included questions regarding sex, age, educational level, number of people living in the household, current studying, and current working status (essential/frontline worker). Age groups were dichotomized according to the median age of the sample (62) into age groups 18–61 and 62 and over.

Questions related to COVID-19 experience included “Have you been under quarantine (self-isolation) due to the COVID-19 outbreak?” “Did a health professional formally diagnose you with COVID-19?” and “Have you lost a significant one during the COVID-19 pandemic?” Possible answers were yes/no.

Lifestyle behaviors were assessed using the Short Multidimensional Inventory Lifestyle Evaluation—Confinement (SMILE-C), which assesses the extent of one’s healthy lifestyle behaviors during the previous 30 days.^24^ The SMILE-C scale comprises 27 items evaluating 7 different lifestyle domains: Diet and nutrition, substance abuse, physical activity, stress management, restorative sleep, social support, and environmental exposures. Response options are measured on a 4-point Likert scale (always, often, seldom, and never), resulting in a maximum possible score of 108. Higher scores indicated healthier lifestyle patterns. After the questions of each domain, an additional question exploring the perceived change in lifestyle patterns during the pandemic as compared to previous, regular behaviors of participants, was added, with possible answer choices of “Completely,” “Moderately,” “Slightly,” and “Not at all.” For the purposes of statistical analysis, the responses “Completely” and “Moderately” were combined as a “Yes” answer, and the responses “Slightly” and “Not at all” were combined as a “No” answer. The additional questions regarding change in lifestyle patterns during the pandemic were not included as part of the SMILE-C assessment or the SMILE-C scoring; they were considered as individual and independent questions in addition to the assessment. Self-rated health was evaluated using the question “How would you rate your health in general?” with possible answer choices of “Very bad,” “Bad,” “Regular,” “Good,” and “Very good.” The responses “Good” and “Very Good” were combined for the analysis.

Previously diagnosed conditions were assessed using the question “In the last 12 months, have you been diagnosed by a medical doctor or health professional, or received treatment for any of the following conditions?” The investigated health problems were combined into chronic diseases, mental health disorders, and infectious diseases. History of chronic diseases included a diagnosis or treatment in the past 12 months of any one or more of the following: diabetes, heart disease, hypertension, asthma, anemia, bronchitis, cancer, cirrhosis, and renal disease. Positive mental health disorders screening included a diagnosis or treatment of depression, anxiety, schizophrenia, bipolar disorder, and/or anorexia/bulimia nervosa. For infectious diseases, we combined cases of participants diagnosed or treated for HIV/AIDS and/or tuberculosis in the past 12 months.

### Outcomes

Two outcomes were considered: positive screening for depression and positive screening for anxiety. Depression was screened using the Patient Health Questionnaire-2 (PHQ-2; cut-off ≥ 3), and anxiety was screened using the Generalized Anxiety Disorder 7-item (GAD-7; cut-off ≥ 10).

### Statistical Analysis

The statistical analysis was conducted using the IBM® SPSS® 27.^25^ All variables, with the exception of SMILE-C scores, were categorized and analyzed using chi-square tests between the respective outcome groups. In addition, we calculated the prevalence ratio (PR) for the significant associations between independent factors and symptoms of depression and anxiety using Epi Info^TM^. Due to the normal distribution of SMILE-C scores, the groups were compared using a student t-test for independent samples. Box plot graphs of the analyses were created using Prisma GraphPad 6.0. Then, multivariate logistic regression analyses were performed to reach the final models for depression and anxiety screening as the 2 outcomes of interest. The analyses included as independent factors those variables that presented an association of *P* < .20 in the bivariate analysis. The final models were reached using a manual stepwise removal of each non-statistically significant variable. Statistical significance was set at *P <* .05.

## Results

A total of 471 participants completed the online web survey. Of those, 62 participants were excluded because they had already completed the survey previously, 3 participants were excluded because they lived outside of Canada, and 2 participants were excluded for being under the age of 18. The final sample size included 404 participants, of which most were female (83.9%), with median age of 62 (IQR: 50–69), had a college/university diploma (76.8%), and were currently unemployed (63.9%). The sample characteristics are described in Table 1.

**Table 1.** Sociodemographic and Clinical Characteristics of the Total Sample.

	Total (n = 404)
Sex	
Female	339 (83.9%)
Male	65 (16.1%)
Age	
18–61	196 (48.5%)
62 or more	208 (51.5%)
Educational attainment	
Elementary/high school	94 (23.3%)
College/university	258 (63.9%)
Graduate school	52 (12.9%)
People in the household	
1	106 (26.2%)
2 or 3	236 (58.4%)
4 to 9	61 (15.1%)
Working	
No	258 (63.9%)
Yes	127 (31.4%)
Lost job during the pandemic	19 (4.7%)
Essential worker (yes)	33 (8.2%)
Frontline worker (yes)	15 (3.7%)
Studying (yes)	42 (10.4%)
Self-isolated (yes)	60 (14.9%)
Lost someone in the pandemic (yes)	22 (5.4%)
Chronic disease*	173 (42.8%)
Mental health disorder*	111 (27.4%)
Infectious disease*	2 (.5%)
Self-rated health (good/very good)	248 (61.4%)
Changed dietary habits during the pandemic (yes)	129 (31.9%)
Changed substance use habits during the pandemic (yes)	27 (6.7%)
Changed physical activity routine during the pandemic (yes)	202 (50.0%)
Changed stress management strategies during the pandemic (yes)	142 (35.1%)
Changed sleep pattern during the pandemic (yes)	111 (27.5%)
Changes in social support during the pandemic (yes)	191 (47.3%)
Changed indoor/outdoor time pattern during the pandemic (yes)	233 (57.7%)
SMILE-C (mean, S.D.)	80.65 (S.D. 8.53)

*= Yes, diagnosed/treated within the last 12 months

The prevalence of positive screenings among the total sample was 24.3% for depression and 20.5% for anxiety, while 15.5% of the sample had positive screenings for both depression and anxiety. Table 2 describes the sociodemographic and clinical characteristics of the sample across the groups of screening for depression. There was an association between age and symptoms of depression in our sample (*P* < .001), where individuals aged 18–61 years were 2.21 times (PR: 2.21 [95% CI: 1.51–3.22]) more likely to present symptoms of depression than older individuals (62 years old or older). There was also a statistically significant association between employment status and screening for depression in our sample (*P* = .006). We conducted an additional analysis comparing individuals who were currently working and those not working, and found that people currently working were 1.63 times (PR: 1.63 [95% CI: 1.14–2.33]) more likely to present symptoms of depression than people that were not working (*P* = .008). Moreover, when comparing individuals who had lost their job during the pandemic with people currently not working, we found that people who had lost their job during the pandemic were 2.17 times (PR: 2.17 [95% CI: 1.21–3.90]) more likely to have symptoms of depression than people that were not working (*P* = .019). There was no difference between groups (positive/negative screening for depression) when comparing people who had lost their job during the pandemic and people that were currently working. There was an association between a previous mental health diagnosis/treatment within the past 12 months and symptoms of depression in our sample (*P* < .001), where people with a previous diagnosis were 2.15 times (PR: 2.15 [95% CI: 1.53–3.03]) more likely to present symptoms of depression than people without a previous mental health diagnosis. We also found an association between SRH and symptoms of depression (*P* = .001), where people that rated their health as bad/very bad were 1.83 times (PR: 1.83 [95% CI: 1.30–2.58]) more likely to present symptoms of depression than those who rated it as “very good/good.” Furthermore, individuals who presented complete or moderate changes in their lifestyle patterns were more likely to present symptoms of depression in comparison to people with slight changes or no changes are all in their patterns. This included changes in dietary patterns (PR: 1.60 [95% CI: 1.13–2.25], *P* = .008), substance use (PR: 1.97 [95% CI: 1.24–3.13], *P* = .011), restorative sleep (PR: 1.89 [95% CI: 1.35–2.65], *P* < .001), and social support (PR: 2.13 [95% CI: 1.47–3.09], *P* < .001).

**Table 2.** Sociodemographic and Clinical Variables based on Presence of Depression.

	Depression Screen		*P*-value
	Yes (n = 97)	No (n = 302)	
Sex			
Female	77 (79.4%)	259 (85.8%)	.134
Male	20 (20.6%)	43 (14.2%)	
Age			
18–61	66 (68.0%)	130 (43.0%)	< .001
62 or more	31 (32.0%)	172 (57.0%)	
Educational attainment			
Elementary/high school	23 (23.7%)	69 (22.8%)	.817
College/university	60 (61.9%)	196 (64.9%)	
Graduate school	14 (14.4%)	37 (12.3%)	
People in the household			
1	29 (29.9%)	74 (24.6%)	.370
2 or 3	57 (58.8%)	178 (59.1%)	
4 to 9	11 (11.3%)	49 (16.3%)	
Working			
No	49 (50.5%)	204 (67.5%)	.006
Yes	40 (41.2%)	87 (28.8%)	
Lost job during the pandemic	8 (8.2%)	11 (3.6%)	
Studying (yes)	11 (11.3%)	31 (10.3%)	.764
Self-isolated (yes)	16 (16.7%)	43 (14.2%)	.560
Lost someone in the pandemic (yes)	7 (7.2%)	13 (4.3%)	.253
Chronic disease*	43 (44.3%)	129 (42.7%)	.780
Mental health disorder*	43 (44.3%)	67 (22.2%)	< .001
Infectious disease*	0	2 (.7%)	1.000
Self-rated health (good/very good)	45 (46.4%)	199 (65.9%)	.001
Changed dietary habits during the pandemic (yes)	42 (43.3%)	87 (28.8%)	.008
Changed substance use habits during the pandemic (yes)	12 (12.4%)	15 (5.0%)	.011
Changed physical activity routine during the pandemic (yes)	56 (57.7%)	142 (47.0%)	.061
Changed stress management strategies during the pandemic (yes)	35 (36.1%)	105 (34.8%)	.795
Changed sleep pattern during the pandemic (yes)	40 (41.2%)	68 (22.5%)	< .001
Changes in social support during the pandemic (yes)	63 (64.9%)	125 (41.4%)	<.001
Changed indoor/outdoor time pattern during the pandemic (yes)	64 (66.0%)	167 (55.3%)	.064
SMILE-C (mean, S.D.)	73.65 (S.D.: 8.12)	82.85 (S.D.: 7.28)	< .001

*= Yes, diagnosed/treated within the last 12 months.

Table 3 describes the sociodemographic and clinical characteristics of the sample across the groups of screening for anxiety. Similarly, there was an association between age and symptoms of anxiety in our sample (*P* < .001), where individuals aged 18–61 years were 2.56 times (PR: 2.56 [95% CI: 1.64–3.97]) more likely to present symptoms of anxiety than older individuals (62 and over). There was also a statistically significant association between employment status and screening for anxiety in our sample (*P* = .022). Additional analyses revealed that when comparing individuals who had lost their job during the pandemic with people currently not working, people who had lost their job during the pandemic were 2.42 times (PR: 2.42 [95% CI: 1.29–4.55]) more likely to present symptoms of anxiety than people who were not working (*P* = .019). There were no differences between groups (negative/positive screening for anxiety) when comparing people who were currently working and those not working, as well as the comparison between people who had lost their job during the pandemic and people currently working. There was an association between a previous mental health diagnosis/treatment within the past 12 months and symptoms of anxiety in our sample (*P* < .001), where people with a previous diagnosis were 3.21 times (PR: 3.21 [95% CI: 2.17–4.74]) more likely to present symptoms of anxiety than people without a previous mental health diagnosis. We also found an association between SRH and symptoms of anxiety (*P* = .002), where people that rated their health as bad/very bad were 1.85 times (PR: 1.85 [95% CI: 1.25–2.73]) more likely to present symptoms of anxiety than those who rated it as “very good/good.” Furthermore, individuals who presented complete or moderate changes in their lifestyle patterns were more likely to present symptoms of anxiety in comparison to people with slight changes or no changes are all in their patterns. This included changes in dietary patterns (PR: 1.55 [95% CI: 1.05–2.29], *P* = .029), restorative sleep (PR: 2.42 [95% CI: 1.66–3.54], *P* < .001), and social support (PR: 1.96 [95% CI: 1.30–2.96], *P* < .001).

**Table 3.** Sociodemographic and Clinical Variables based on Presence of Anxiety.

	Anxiety Screen		*P*-value
	Yes (n = 80)	No (n = 310)	
Sex			
Female	64 (80.0%)	264 (85.2%)	.260
Male	16 (20.0%)	46 (14.8%)	
Age			
18–61	57 (71.3%)	135 (43.5%)	< .001
62 or more	23 (28.8%)	175 (56.5%)	
Educational attainment			
Elementary/high school	25 (31.3%)	65 (21.0%)	.136
College/university	47 (58.8%)	203 (65.5%)	
Graduate school	8 (10.0%)	42 (13.5%)	
People in the household			
1	16 (20.0%)	83 (26.9%)	.231
2 or 3	54 (67.5%)	176 (57.0%)	
4 to 9	10 (12.5%)	50 (16.2%)	
Working			
No	42 (52.5%)	205 (66.1%)	.022
Yes	31 (38.8%)	95 (30.6%)	
Lost job during the pandemic	7 (8.8%)	10 (3.2%)	
Studying (yes)	9 (11.3%)	32 (10.3%)	.809
Self-isolated (yes)	13 (16.5%)	46 (14.8%)	.721
Lost someone in the pandemic (yes)	3 (3.8%)	18 (5.8%)	.654
Chronic disease*	36 (45.0%)	130 (41.9%)	.621
Mental health disorder*	43 (53.8%)	65 (21.0%)	< .001
Infectious disease*	1 (1.3%)	1 (.3%)	.875
Self-rated health (good/Very good)	37 (46.3%)	202 (65.2%)	.002
Changed dietary habits during the pandemic (yes)	34 (42.5%)	92 (29.7%)	.029
Changed substance use habits during the pandemic (yes)	9 (11.3%)	17 (5.5%)	.063
Changed physical activity routine during the pandemic (yes)	40 (50.0%)	154 (49.7%)	.920
Changed stress management strategies during the pandemic (yes)	25 (31.3%)	113 (36.5%)	.425
Changed sleep pattern during the pandemic (yes)	38 (47.5%)	68 (21.9%)	< .001
Changes in social support during the pandemic (yes)	50 (62.5%)	132 (42.6%)	.001
Changed indoor/outdoor time pattern during the pandemic (yes)	48 (60.0%)	175 (56.5%)	.567
SMILE-C (mean, S.D.)	73.63 (S.D. 8.25)	82.46 (S.D. 7.63)	< .001

*= Yes, diagnosed/treated within the last 12 months.

**Figure 1.**
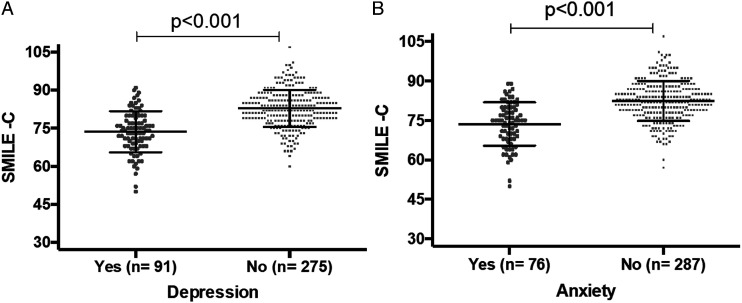
(A) Mean SMILE-C scores categorized based on screening for depression. Legend: SMILE-C: Short Multidimensional Inventory Lifestyle Evaluation—Confinement. (B) Mean SMILE-C scores categorized based on screening for anxiety. Legend: SMILE-C: Short Multidimensional Inventory Lifestyle Evaluation—Confinement.

Individuals with symptoms of depression showed significantly lower SMILE-C scores (mean: 73.65; S.D.: 8.12) as compared to individuals without symptoms of depression (mean: 82.85; S.D.: 7.28, *P* < .001) (Figure 1A). The difference in scores between the groups indicates significantly unhealthier lifestyle behaviors in people with symptoms of depression during the COVID-19 pandemic.

Similarly, participants with symptoms of anxiety also had lower scores in the SMILE-C (mean: 73.63; S.D.: 8.25) as compared to participants without symptoms of anxiety (mean: 82.46; S.D.: 7.63, *P* < .001) (Figure 1B). This difference suggests significantly unhealthier lifestyle behaviors in people with symptoms of anxiety during the COVID-19 confinement period.

Table 4 presents the final multivariate model for depression, while Table 5 presents the final multivariate model for anxiety. In the final multivariate models for depression and anxiety, being between the ages of 18–61 was associated with an increased prevalence of depression (adjusted odds ratio (OR): 2.64; 95% CI: 1.46–4.76) as well as anxiety (OR: 2.57; 95% CI: 1.34–4.94), as compared to individuals aged 62 or older. Furthermore, complete or moderate changes in social support during the pandemic were associated with increased likelihood for both depression (OR: 2.91; 95% CI: 1.61–5.24), and anxiety (OR: 2.11; 95% CI: 1.11–4.01), as compared to mild or no changes in social support during the pandemic. A mental health diagnosis within the past 12 months increased the likelihood for anxiety (OR: 2.71; 95% CI: 1.45–5.05), as did complete or moderate changes in sleep patterns (OR: 2.31; 95% CI: 1.22–4.38). Lower SMILE-C scores remained associated with symptoms of depression (OR: .86; CI: .82–.89) and anxiety (OR: .89; CI: .85–.92) in the final multivariate models.

**Table 4.** Multivariable Logistic Regression Examining the Associations between Depression and Sociodemographic Variables (n = 365).

Variable		Depression		*P*-value
		Multivariable Adjusted		
		OR	95% CI	
Age				
	62 or more	1		
	18 to 61	2.64	1.464 to 4.762	.001
Changes in social support during the pandemic				
	No	1		
	Yes	2.909	1.613 to 5.245	< .001
SMILE-C		.859	.824 to .896	< .001

OR: odds ratio; CI: confidence interval.

**Table 5.** Multivariable Logistic Regression Examining the Associations between Anxiety and Sociodemographic Variables (n = 355).

Variable		Anxiety		*P*-value
		Multivariable Adjusted		
		OR	95% CI	
Age				
	62 or more	1		
	18 to 61	2.575	1.342 to 4.940	.004
Mental health disorder*				
	No	1		
	Yes	2.707	1.453 to 5.046	.002
Changed sleep pattern during the pandemic				
	No	1		
	Yes	2.311	1.219 to 4.382	.010
Changes in social support during the pandemic (yes)				
	No	1		
	Yes	2.112	1.113 to 4.007	.022
SMILE-C		.887	.851 to .925	< .001

OR: odds ratio; CI: confidence interval. * = Yes, diagnosed/treated within the last 12 months.

## Discussion

To the best of our knowledge, this is the first study indicating an association between lifestyle behaviors and mental health symptoms during the COVID-19 confinement period in Canada, where people with symptoms of depression or anxiety engaged in more unhealthy lifestyle patterns than individuals with a negative screen for these symptoms. Findings from our study are in line with reports previously discussed, highlighting the relationship between different lifestyle domains, such as diet, physical activity, social relationships, and sleep quality, and symptoms of common mental health problems,^11,26-28^ which have been further emphasized during the COVID-19 pandemic around the world.^15,16^

In correspondence to our findings, unhealthy lifestyle changes measured using the SMILE-C scale were also associated with depression and anxiety among essential workers in Brazil and Spain at the early stages of the pandemic.^18^ Recent evidence suggests that the bidirectional relationship between lifestyle behaviors and mental health that has emerged during the pandemic will persist over a long period, as overall lifestyle quality worsened among Spanish citizens as the confinement period continued.^29^ Findings from our study are in line with these reports, highlighting the association between unhealthy lifestyle behaviors and mental health symptoms during the COVID-19 lockdown in Canada. Generally, adults that had experienced complete or moderate changes in their sleep and social support patterns, and reported overall unhealthier lifestyle behaviors during the confinement period were more likely to present symptoms of depression and anxiety during the COVID-19 pandemic.

Furthermore, our findings suggest an association between some sociodemographic factors and symptoms of depression and anxiety. In our sample, we found that older individuals were less likely to report symptoms of depression and anxiety than younger adults, a trend which has been suggested by reports from other countries during the pandemic.^30,31^ It is possible that younger adults have experienced greater changes in their everyday life during the COVID-19 pandemic, leading to psychological distress and feelings of loneliness, while older adults may have had fewer pandemic-based life disruptions and may be more psychologically resilient in the face of stressful events.^32,33^ In addition, we found that individuals who reported changes in their social support during the pandemic were more likely to present with symptoms of depression and anxiety. With the social isolation measures that have been put in place since the onset of the COVID-19 pandemic, many people have experienced loneliness and have had to adjust their social communication methods, leading to elevated levels of mental health symptoms.^33^ Indeed, reports from the early stages of the pandemic found an inverse relationship between social support and symptoms of depression and anxiety,^34^ highlighting the impact of the isolation measures on the global mental health burden. We also found that individuals who reported changes in their sleep patterns were more likely to present with symptoms of anxiety during the pandemic in Canada. The association between poor sleep quality and anxiety is well-established^35^ and has been documented during the COVID-19 pandemic in Canada and worldwide.^36,37^ As the pandemic progresses and affects people around the globe, more resources will be needed to help people with sleep disturbances. Furthermore, individuals with a previous mental health diagnosis or treatment within the past 12 months were more likely to have a positive screen for anxiety at the time of the survey. The ongoing concerns and challenges related to the pandemic, as well as the decline in access to care at the beginning of the pandemic, may have led to more severe psychological impacts in people with pre-existing psychiatric conditions than those without a pre-existing condition.^38^ Just as we have seen a rise in the incidence rates of anxiety during the pandemic, individuals with pre-existing anxiety in Canada have experienced clinically significant worsening of these symptoms, suggesting the need for urgent interventions in this population.^38^

The frequency of unhealthy lifestyle behaviors in this study was detected using the SMILE-C scale, which is an instrument specifically designed to identify unhealthy patterns during the COVID-19 pandemic using a multidimensional approach.^24^ The importance of lifestyle domains in relation to mental health has traditionally been evaluated through separate assessments for each individual domain, such as the Food Frequency Questionnaire,^39^ International Physical Activity Questionnaire,^40^ and the Pittsburgh Sleep Quality Index,^41^ to name a few. However, the fast-growing perspective of LM implies that the combined focus on different lifestyle behaviors could have a greater effect on prevention and treatment of psychiatric symptoms and disorders.^42,43^ Domains included in the SMILE-C scale are considered to be modifiable factors that have the potential to improve both physical and mental well-being, as well as possibly prevent the onset of common mental health disorders.^44^ Considering the sudden, distressing changes of various lifestyle areas during the COVID-19 confinement period, a multidimensional approach to lifestyle assessment could help us better understand the combined effect of various unhealthy behaviors on the appearance of psychological symptoms in the general population.

Findings from this study should be interpreted considering some limitations. The cross-sectional design of the study limits our ability to attribute the symptoms of depression and anxiety to the unhealthy lifestyle behaviors indicated by the SMILE-C. However, an association between these variables is suggested. Also, considering that the collection of data was completed using a web survey and the sample was not probabilistic, any generalization of the results must be made with caution. For instance, our sample consisted predominantly of older adults (median age: 62), unemployed individuals, and females, which is not representative of the general population of Canada and could potentially impact the interpretation of the findings. Lastly, the assessment of psychological symptoms was completed using self-report screening tests such as the PHQ-2 and GAD-7; the replication of this data using a structured clinical interview is encouraged.

## Conclusion

This is the first study to examine the association between lifestyle behaviors and symptoms of common mental disorders during the COVID-19 pandemic in Canada. Our findings revealed an association between self-reported unhealthy lifestyle behaviors and positive screenings for symptoms of depression and anxiety during the COVID-19 lockdown period in Canada. These findings are concurrent with reports from various parts of the world, further demonstrating the global importance of the relationship between lifestyle and mental health challenges during the COVID-19 pandemic. In addition, the multidimensional approach to lifestyle assessments and treatments is gaining attraction and becoming more frequent along with the field of LM. Unhealthy behaviors in different lifestyle domains tend to cluster and have a cumulative effect on the mental well-being of a person^45^; however, most epidemiological studies to date have considered unhealthy behaviors as independent risk factors of mental health disorders. Therefore, it is important to raise awareness and educate the public, as well as healthcare providers, on the significance of cumulative, modifiable, lifestyle factors for population mental health, especially during the ongoing pandemic.^46-48^ Findings from this study highlight the need for developing targeted approaches to promote healthy lifestyles, which could help in reducing the burden of common mental disorders in Canada and worldwide.
